# Dementia in residential care: education intervention trial (DIRECT); protocol for a randomised controlled trial

**DOI:** 10.1186/1745-6215-11-63

**Published:** 2010-05-26

**Authors:** Christopher D Beer, Barbara Horner, Osvaldo P Almeida, Samuel Scherer, Nicola T Lautenschlager, Nick Bretland, Penelope Flett, Frank Schaper, Leon Flicker

**Affiliations:** 1 WA Centre for Health and Ageing, Centre for Medical Research, Perth, Western Australia, Australia; 2 School of Medicine and Pharmacology, University of Western Australia, Australia; 3 Centre for Research on Ageing, Curtin University of Technology, Perth, Western Australia, Australia; 4 School of Psychiatry and Neurosciences, University of Western Australia, Australia; 5 Royal Freemasons Homes of Victoria, Australia; 6 Academic Unit for Psychiatry of Old Age, St Vincent's Health, Department of Psychiatry, University of Melbourne, Australia; 7 Rowethorpe Medical Centre, Bentley, Western Australia, Australia; 8 Brightwater Care Group (Inc), Perth, Western Australia, Australia; 9 Alzheimer's Australia WA Ltd, Perth, Western Australia, Australia

## Abstract

**Background:**

There is scope to improve the quality of life (QOL) of people with dementia living in residential care facilities (RCF). The DIRECT study will determine if delivery of education to General Practitioners (GPs) and care staff improves the quality of life of residential care recipients with cognitive impairment.

**Methods/Design:**

A prospective randomised controlled trial conduced in residential aged care facilities in the metropolitan area of Perth, Western Australia. Participants are care facility residents, aged 65 years and older and with mini-mental state examination scores less than 25. GPs and care facility staff have been independently randomised to intervention or control groups. An education programme, designed to meet the perceived needs of learners, will be delivered to GPs and care staff in the intervention groups. The primary outcome of the study will be quality of life of the people with dementia, measured using the QOL-Alzheimer's Disease Scale (QOL-AD) and Alzheimer Disease Related QOL Scale (ADRQL), 4 weeks and 6 months after the conclusion of the education intervention.

**Results:**

Recruitment of 351 people with dementia, cared for by staff in 39 residential facilities and 55 GPs, was undertaken between May 2007 and July 2008. Collection of baseline data is complete. Education has been delivered to GPs and Care staff between September 2008 and July 2009. Follow- up data collection is underway.

**Discussion:**

The study results will have tangible implications for proprietors, managers and staff from the residential care sector and policy makers. The results have potential to directly benefit the quality of life of both patients and carers.

**Trial registration:**

These trial methods have been prospectively registered (ACTRN12607000417482).

## Background

Many Australians with dementia, nearly half, live in residential care [1]. A large proportion of RCF residents have dementia, the majority with moderate-severe dementia [2-4]. However only a small proportion of beds are dementia-specific, and these have usually been designed to deal with specific behavioural and psychological challenges associated with dementia, such as frequent wandering [1].

Little is known about the subjective experience of people with dementia living in residential care. The available data suggest that people with moderate to severe dementia frequently experience distressing emotions [5]. Thus, there appears to be much scope to improve the experiences of residential care recipients.

Many of the common challenges encountered in caring for people with dementia can be ameliorated. For example behavioural treatments and non pharmacological interventions may reduce sleep and behavioural disturbances [6-8]. Changes to existing models of care, such as implementation of protocols to engage family caregivers, and environmental changes, may be effective in slowing residents' deterioration [9]. Likewise, there are data confirming that high quality care is associated with a lower incidence of depression [10].

Education and training appear to be effective in improving care outcomes. The introduction of dementia programmes in mainstream Australian hostels is thought to have been effective in delaying transfer to higher level care and in improving quality of life [11]. Education of nursing assistants and ancillary staff can improve their attitudes and knowledge regarding end of life care [12]. Training and support for nursing home staff is effective in reducing the proportion of residents with dementia who are prescribed neuroleptic medications (average reduction in neuroleptic use 19.1%; 95% confidence interval 0.5% to 37.7%) [13]. A marked reduction in psychotropic prescribing in Sydney nursing homes occurred in line with educational interventions and publicity regarding inappropriate medication use [14].

Cross sectional data suggest that staff training and specialisation are associated with improvements in the quality of life of people with dementia living in residential care [15]. However our data confirm that, despite staff and GPs working in residential care frequently participating in education and generally perceiving their knowledge as good, perceived educational needs persist [16]. The most effective way to translate the available evidence to practice is not clear and there is a paucity of prospective data to guide practice. The Dementia in Residential Care: education intervention Trial ("DIRECT") will determine if an educational intervention, designed in light of the perceived needs of learners, will enhance the quality if life of care recipients.

## Methods

### Study Design and Setting

DIRECT is a prospective randomised controlled trial conducted in residential aged care facilities of the metropolitan area of Perth, Western Australia.

### Participants

Inclusion criteria are i) permanent resident of a low-level or high-level residential care facility; ii) greater than 65 years of age; and iii) MMSE score ≤24. Exclusion criteria are i) participant's general practitioner works at more than one facility participating in the trial; ii) subject is identified by facility as medically unstable or as suffering delirium, or in the terminal stages of a co-morbid illness; iii) subject unable to participate in assessment instruments in English.

All residential care facilities (RCFs) in the Perth metropolitan area (n = 184) were sent information packages regarding the DIRECT Study. Of those, 36 agreed to participate. Participating RCFs compiled a list of residents to be screened for study participation. GPs working at the facility and residents meeting the inclusion criteria were invited to participate in the study. Study staff made second attempts to recruit GPs if residents they cared for had agreed to study participation, but the GP had not responded to the initial contact.

### Randomisation

Randomisation will be carried out in a factorial fashion. That is care facilities and GPs will be independently randomised to intervention or control groups. Allocation will be by a centrally held computer generated randomisation table, managed by an independent statistician.

### Intervention

A detailed qualitative research study has been undertaken to determine the perceived needs of learners [16]. This informed development of an educational package which was delivered to GPs, clinical and direct care staff between September 2008 and July 2009. The main topics of the educational programs were:

• communication with residents and family members

• personal care and activities

• positive values

• behaviours of concern

• pain management

• dementia, depression and delirium

• effective working between GPs and RCF.

The GP education program consisted of five modules, delivered during three evening sessions. A fourth, reflective, session was also held. The sessions used case scenarios from DVDs and role plays with volunteers and professional actors to stimulate participation, consistent with adult learning principles. They were facilitated by study staff and one or two of the authors. A self-directed learning package (DVD of the first three sessions plus supporting materials), and a reflection session, were offered to GPs not attending face-to-face workshops. The GP program was approved for 40 Category 1 Continuing Professional Development points for the 2008-2010 triennium. The RCF education intervention comprised 27 brief modules which were delivered on-site at each facility by one of two educators. This format was chosen to facilitate flexibility in delivery of the program. Each of the 27 lessons was in half hour blocks which could be built into sessions of varying lengths of time. Education sessions ranged from 1 hr blocks to full 7.5 hr days.

### Outcomes

Outcomes will be measured by blinded research assistants. Research assistants have been trained in the standard administration of assessment tools and adequate inter-rater reliability established for the QOL-AD [17].

The primary outcome of the study is the quality of life of the residents, measured using the (Alzheimer Disease Related QOL Scale (ADRQOL) [18] which relies on caregiver interview, and the QOL-Alzheimer's Disease Scale (QOL-AD) modified for use in long--term care settings [19,20] which utilizes self report and caregiver interview. Secondary outcomes include behavioural and psychologic symptoms of dementia (measured with the Neuropsychiatric Inventory- NH version [21]), use of restraint, and pain (measured using the Brief Pain Inventory modified verbal form [22]). Outcomes will be assessed at 4 weeks and 6 months after the conclusion of the educational intervention.

### Sample size and statistical analysis

Training for care providers has been associated with a 0.4 point mean fall in QOL scores (SD 7.6) over 6 months, while QOL fell by a mean of 3.5 points (SD 7.9) in facilities that did not train care providers [10]. We have powered the present study to detect an effect of at least this magnitude. We would need 100 subjects in each row of the two by two factorial table (total number 200) to have 0.8 power to detect a main effect of this magnitude (alpha = 0.05; two sided). Loss of subjects to follow up, based on our previous experience, was anticipated to be in the order of 30% per annum in this population [23]. We used an estimated intra-class correlation of 0.05 (to account for clustering) and estimated cluster size of 9, resulting in an inflation factor of 1.4. We thus expected to have to enrol 364 subjects in forty clusters to achieve adequate numbers of completing subjects. This estimate of the intraclass correlation was conservative, given that intraclass correlation coefficients are typically smaller than 0.02 [24]. The power calculation was reviewed prior to closing recruitment, because actual cluster size (which influences the required number of participants) can be difficult to predict. Having recruited 67 clusters with an average cluster size of 5.2, it was confirmed that, because cluster size was smaller than anticipated, recruitment could be closed. Analysis will be by "intention to treat" (that is, according to randomisation, rather than participation in education). Statistical analysis will be conducted using multilevel mixed-effects linear regression in Stata version 11.0 (StataCorp, College Station, Texas). The effect of clustering by both facility and GP will be accounted for by treating the facility and GP as random effects with GP nested within facility. For each outcome analysis, a model containing the GP intervention, the facility intervention, and the baseline values of outcome variables will be used to estimate the marginal effect of each intervention. Next, the confounding effects of other covariates will be examined by comparing the adjusted and unadjusted intervention effects. Any covariates that produce clinically important changes in the intervention effect estimates, and are therefore demonstrably confounding these effects, will be retained in the model. Secondary analyses will be conducted to test the significance of any interaction between the facility and GP interventions. The results reported will include the estimated mean of the outcome variable in each arm of both interventions and for each intervention, the mean difference in outcome between the intervention and control arms. The results of the secondary analysis of the interaction between interventions will also be presented. All results will be presented with their associated 95% confidence intervals.

### Ethics Approval and Trial Registration

The Human Research Ethics Committee at the University of Western Australia approved this study (RA 4/1/1685). All GPs and RCF provided written agreement to participate in the study. Structured written and verbal consent procedures were used by research staff when approaching residents with cognitive impairment. The assent of "next of kin" was required for participation of people with cognitive impairment who were unable to provide informed consent. This trial was registered (ACTRN12607000417482) on 17/08/2007.

## Results

**Figure 1:**
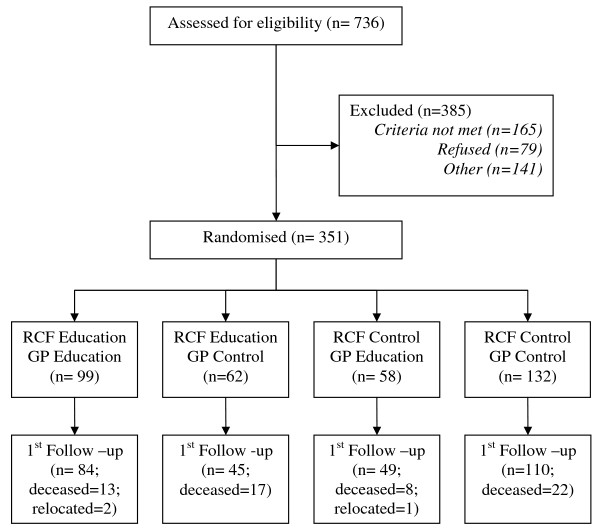
**Study Flow Chart**.

Recruitment of 350 people with dementia, living in 39 care facilities and cared for by 55 GPs is now complete. Collection of baseline data is complete. Education has been delivered to GPs and Care staff between September 2008 and July 2009. First follow-up data collection has been completed (Figure 1). Analysis will be completed at the conclusion of second follow-up assessments after but remains blinded as second follow- up data collection is ongoing. Baseline data of participants completing the first follow-up assessment, and those lost to follow-up at the first follow-up assessment are summarised in Table 1.

**Table 1:** Participant Characteristics.

Variable	First Follow-up Completed n = 288	Lost at First Follow Up n = 63	p
Age	85.1 ± 7.9	86.1 ± 7.8	0.367

Gender (Male)	58 (20%)	28 (44%)	0.000

MMSE	14 (6-20)	12 (8-18)	0.326

Weight (kg)	62.1 ± 14.0	63.6 ± 14.9	0.434

Self Rated QOL-AD	41.1 ± 6.00	42.3 ± 5.7	0.381

Staff Rated QOL-AD	32.5 ± 7.6	30.2 ± 6.5	0.017

NOK Rated QOL-AD	32.4 ± 8.1	32.4 ± 9.1	0.972

Staff Rated ADRQL	73.5 ± 16.5	69.7 ± 15.4	0.096

NOK Rated ADRQL	74.8 ± 14.5	75.2 ± 15.5	0.893

Number of medications	9 (7-12)	11 (8-13)	0.047

10 Item NPI	13 (4-29)	12 (4-26)	0.927

12 Item NPI	14 (5-32)	16 (6-27)	0.860

10 Item NPI Distress	4 (0-10)	3 (0-8)	0.431

12 Item NPI Distress	4 (1-11)	4 (1-8)	0.670

Values are mean ± SD or median (IQR); MMSE = mini-mental stare examination score, NOK= next of kin, ADRQOL = Alzheimer Disease Related QOL Scale, QOL-AD = QOL-Alzheimer's Disease Scale modified for use in long--term care settings, NPI = Neuropsychiatric Inventory- NH version [21]

## Discussion

This project will use a pragmatic research agenda to address highly relevant research questions. It is anticipated that the results, positive or negative, will be of value to policy makers and stakeholders from the residential care industry and peak community and general practice bodies. The study results will have tangible implications for proprietors, managers and staff from the residential care sector and policy makers. Through improvement in care delivery, the project has potential to directly improve the quality of life of people with dementia living in residential care facilities and their carers.

The study design has several strengths. An important component of this study's design is an intervention based on a systematic study of learner's perceived needs. If effective the intervention will be easily transferable to services outside of research projects. The choice of quality of life, measured from multiple points of view, as the primary outcome is also a strength of the study. The present study will help us to understand to what extent education delivered to GPs and staff results in a measurable change in the wellbeing of people with dementia. There is no universally accepted standard for measuring QOL of people with dementia. This reflects the difficulty of establishing a theoretical framework to measure a concept which is essentially subjective. In addition, the logistical barriers to determining the views of people with cognitive impairment, and the potential biases associated with reliance informants to determine QOL, make reliable measurements of QOL difficult. The available data suggest that people with moderate to severe dementia frequently retain degrees of awareness [25]. Many people with dementia remain able to report their met and unmet needs and reliably rate their quality of life [19,26]. Assessments made by people themselves may differ from those of informants [27]. We have thus chosen to utilise a combination of self and informant report scales. Inclusion of early and delayed follow-up will allow us to determine if there are sustained effects of the intervention on quality of life of residents.

Our study design is also strengthened by the explicit inclusion of both GPs and care staff. This reflects clinical situations in which facility staff and GPs both make important contributions to the care of residents. However inclusion of both GPs and RCF staff produces several challenges. The study design does not exclude contamination between intervention and control groups, nor is the study powered to detect an interaction between the GP and RCF staff interventions. The resultant study design (where GPs and RCF are independently randomised) is a pragmatic solution. It reflects what may occur in practice, given the current separation of educational programs for doctors and the aged care industry in Australia.

There are several further limitations to the study design. Our inclusion criteria did not require a confirmed clinical diagnosis of dementia. The exclusion criteria attempted to reduce the chances of participation by people with other common diagnoses such as delirium. Because dementia is under-recognised we felt that requiring an existing diagnosis of dementia would introduce a bias. Confirmation of a dementia diagnosis by a study clinician would have been optimal, but was not considered feasible. As with any randomised trial, volunteer bias may limit generalisability of the findings. Care facilities and GPs choosing to participate in the study may tend to be those who already hold positive attitudes toward, and are seeking additional, education. Use of an intention to treat analysis (that is, including GPs and care staff who did not participate in education) will help to maximise generalisability of the results to real world situations, where uptake of educational interventions is likely to be incomplete.

## Conclusion

Many people with dementia require residential care. There is an imperative to improve the quality of life of people with dementia living in Residential Care Facilities. However, the most effective way to translate knowledge regarding the components of high quality care into practice is uncertain. Educational interventions appear to be effective. This study will determine whether delivery of an educational intervention to GPs and care staff, will improve the quality of life of care recipients. A number of unique features mean that the results of this trial are likely to be generalisable.

## Competing interests

The authors declare that they have no competing interests.

## Authors' contributions

CDB, OPA, LF and NTL conceived the study. BH, SS, PF and FS contributed to study design. CDB drafted the manuscript. All authors contributed to critical review of the manuscript.
